# The Impact of Periodontal Instrumentation on Enamel and Cementum: A Narrative Review

**DOI:** 10.3390/dj14050259

**Published:** 2026-04-30

**Authors:** Maike Lodigkeit, Mariusz Lipski, Laurentia Schuster, Till Dammaschke, Włodzimierz Dura, Martyna Mochol, Małgorzata Mazurek-Mochol

**Affiliations:** 1 Department of Periodontology, Pomeranian Medical University in Szczecin, Powstańców Wlkp 72, 70-111 Szczecin, Poland; 2 Department of Preclinical Conservative Dentistry and Preclinical Endodontics, Pomeranian Medical University in Szczecin, Powstańców Wlkp 72, 70-111 Szczecin, Poland; 3 Department of Periodontology and Operative Dentistry, University of Münster, 48149 Münster, Germany

**Keywords:** periodontal instrumentation, periodontal health, plaque, calculus

## Abstract

**Objectives:** Achieving a smooth tooth surface following periodontal instrumentation is critical for maintaining periodontal health and minimizing plaque and calculus reaccumulation. This narrative review aimed to synthesize preclinical laboratory-based evidence regarding the effects of periodontal instrumentation techniques on enamel and cementum surface roughness and hard-tissue loss. **Methods:** A focused literature search was conducted using the PubMed database to identify relevant in vitro and in vitro/in vivo hybrid studies published within the last 15 years. This review focused on ultrasonic scaling, hand instrumentation, and air polishing of human teeth during periodontal treatment. **Results:** Periodontal instrumentation was associated with surface alterations of enamel and cementum, with the extent of these changes depending on instrumentation parameters. Manual instrumentation was generally associated with greater surface irregularities and increased cementum removal, whereas ultrasonic scaling tended to produce more uniform surface characteristics. However, outcomes varied depending on instrumentation parameters. Air-polishing systems were described as less abrasive, particularly in biofilm management. **Conclusions:** Within the limitations of predominantly in vitro evidence, periodontal instrumentation appears to alter dental hard tissues to varying degrees depending on the technique and application. The clinical relevance of these findings remains uncertain, as the evidence was primarily laboratory-based and may not fully reflect clinical conditions or predict long-term outcomes.

## 1. Introduction

Periodontal diseases remain among the most common chronic inflammatory diseases affecting adult populations worldwide. The primary etiological factor is dental biofilm, and when left untreated, it can lead to gingival inflammation, attachment tissue breakdown, and progressive alveolar bone loss. Mechanical disruption and removal of biofilm and mineralized deposits are, therefore, the foundation of preventive and therapeutic periodontal care. Periodontal instrumentation, including hand scaling, ultrasonic debridement, and air-abrasive biofilm removal, is essential for achieving periodontal stability and long-term tooth retention [1,2].

Traditionally, root planing has involved the removal of the affected cementum to eliminate endotoxin within the contaminated cementum. Current studies question the traditional approach of root planing and emphasize the preservation of root structure and biologically active cementum wherever possible. Cementum plays a regulatory role in periodontal healing and attachment by influencing fibroblast behaviour and connective tissue reattachment. Furthermore, excessive removal of root cementum can eliminate bioactive proteins and growth factors essential for periodontal regeneration and fibrous attachment. Therefore, excessive removal may impair the regenerative potential of periodontal connective tissue [3,4,5].

Effective periodontal instrumentation reduces microbial load, disrupts pathogenic biofilms, and creates a smooth root surface, which promotes soft-tissue healing. However, it should be noted that mechanical debridement of mineralized tooth tissue is associated with some degree of hard tissue alteration, ranging from minor roughness changes to measurable substance loss [6]. Increased surface roughness has been associated with enhanced bacterial adhesion and plaque retention, which interferes with periodontal healing [7].

Enamel and cementum are most directly exposed to mechanical instrumentation during both supragingival and subgingival debridement procedures [8]. Although enamel is the most mineralized tissue in the human body, it can be damaged during professional debridement. Instrumentation may induce surface scratches, micro-grooves, and increased surface roughness [9,10]. Cementum is considerably softer and thinner than enamel. Therefore, it is more vulnerable to instrumentation trauma. Preservation of this tissue is crucial, given its role in periodontal attachment and healing [3,4].

Other clinical consequences of dental hard tissue alterations may also include hypersensitivity and loss of marginal integrity of restorations [11,12].

A structured synthesis of this evidence is clinically relevant, as modern periodontal therapy focuses on minimally invasive approaches and long-term preservation of dentition. Consequently, periodontal instrumentation is performed repeatedly throughout a patient’s life [13,14]. Even minor cumulative effects on enamel and cementum may have clinical significance. A clear understanding of these effects may support evidence-based decision-making and highlight the importance of operator technique and training to minimize iatrogenic damage.

While individual in vitro studies have investigated surface alterations and hard-tissue loss, their findings are heterogeneous and sometimes contradictory. No prior narrative or systematic synthesis focusing specifically on alterations in enamel and cementum following periodontal instrumentation was identified. Therefore, this study aimed to provide a narrative synthesis of preclinical laboratory-based evidence regarding the effects of periodontal instrumentation on enamel and cementum, with particular emphasis on surface roughness and hard-tissue loss.

## 2. Methods

### 2.1. Literature Search Strategy

This study was conducted as a focused narrative review. Relevant literature was identified through a search of the PubMed database, which was considered suitable because of its broad coverage of biomedical and dental research [15]. The search was conducted iteratively using combinations of keywords related to instrumentation (“periodontal instrumentation”, “ultrasonic scaling”, “root planing”, “air polishing”, “periodontal debridement”, “hand instrumentation”), substrate (“enamel”, “cementum”) and surface alteration (“surface roughness”, “surface damage”, “loss of enamel/cementum”, “substance loss”). Different terms from each group were combined using Boolean operators.

The selection was limited to studies published in English to ensure accurate interpretation of the included works. A 15-year publication window was applied to focus on contemporary evidence and ensure clinical relevance, given ongoing advancements in periodontal instrumentation technologies and techniques. These limitations may introduce selection and language bias, which should be considered when interpreting the findings. The final literature search for this review was conducted in January 2026.

### 2.2. Study Selection

Publications were included if they investigated the effects of periodontal instrumentation on enamel or cementum in preclinical laboratory-based studies, including in vitro studies on extracted human teeth and in vitro/in vivo hybrid studies, in which instrumentation was performed clinically prior to laboratory analysis. Studies evaluating outcomes such as surface roughness, morphological alterations, or hard-tissue loss and assessing commonly used periodontal instrumentation methods, including ultrasonic scaling, hand instrumentation, and air polishing, were included [16]. The inclusion of human teeth was intended to enhance the clinical relevance of the findings.

Studies were excluded if they involved animal teeth, artificial tooth models, orthodontic instrumentation, restorative procedures, implant surfaces, or non-periodontal instrumentation methods.

Final study selection was limited to publications that could be retrieved in full text to allow a complete evaluation of study methods and outcomes. While this approach ensured that all included studies could be fully evaluated and accessed, it may have introduced selection bias.

### 2.3. Data Extraction and Synthesis

Relevant data from the selected studies were manually extracted and qualitatively summarized by the authors. Extracted information includes the type of instrumentation, study design, evaluated substrate, measurement techniques, and reported outcomes.

To structure the synthesis, studies were grouped according to evaluated substrate (enamel or cementum), type of instrumentation (e.g., hand instrumentation, ultrasonic scaling, air polishing), and outcome measures (surface roughness, morphological alterations, and hard-tissue loss). Due to the heterogeneity of study designs, instrumentation parameters, and measurement techniques, a quantitative synthesis (meta-analysis) was not considered appropriate. Therefore, findings were synthesized narratively, emphasizing common patterns, differences between instrumentation methods, and potential clinical implications. Conclusions were based on the consistency of findings across studies, while differences were interpreted in the context of variations in study design and methodology.

## 3. Results

This review included 16 studies comprising both in vitro and in vitro/in vivo hybrid designs (Table 1). These studies examined the effects of periodontal instrumentation on enamel and cementum surface characteristics, with particular attention to surface roughness and hard-tissue loss. The instrumentation methods investigated included hand instruments, ultrasonic devices with various power settings and tip materials, rotary instruments, polishing systems, and air-polishing systems. Outcomes were evaluated primarily using scanning electron microscopy (SEM), atomic force microscopy (AFM), and profilometric analyses [17,18,19,20,21,22,23,24,25].

### 3.1. Effects on Enamel Surface Topography and Roughness

Instrumentation of enamel consistently resulted in detectable changes on the surface regardless of the method [7,18,19,20]. Ultrasonic and manual scaling both increased enamel roughness, although differences in damage were observed by instrument type, angulation, pressure, and application duration [18].

Stainless-steel ultrasonic scalers produced fine superficial scratches with minimal surface roughness, whereas titanium tips produced deeper, more pronounced defects. Hand scaling with titanium curettes produced the most significant enamel damage. These findings may be attributed to the greater material hardness of titanium and the greater force applied during manual instrumentation [18].

Arefnia et al. (2021) [19] conducted an in vitro study evaluating changes in enamel surface roughness following air polishing, ultrasonic scaling, and hand instrumentation. Results were reported as Roughness depth (Rz), which describes the average of maximum peak-to-valley distances. Each technique was tested alone to allow comparison. Air polishing with a non-abrasive 7 μm powder produced the highest mean roughness depth at 6.22 μm. Manual curettage resulted in an Rz of 5.04 μm, while ultrasonic scaling resulted in an Rz of 3.44 μm. Despite these differences, none of the treatments caused a statistically significant change in Rz compared to untreated enamel in the control group (Rz of 3.0 to 6.0 μm). Overall, the findings indicate that although each instrument produces a distinct surface topography, none significantly alters the natural roughness of enamel when used independently [19].

Yildirim et al. (2021) [7] tested, in an in vitro study, the effects of different scaling and polishing techniques. Enamel surfaces were standardized before treatment with carbide papers of different grit sizes to ensure a uniform baseline and enable comparison of the results. The researchers found that all tested instrumentation techniques (ultrasonic scaling, hand instrumentation, polishing paste, pumice, and air-powder system) significantly increased enamel surface roughness. Specifically, ultrasonic scalers on enamel resulted in the greatest increase in surface roughness. The authors attributed this to the vibrational forces generated by ultrasonic devices, which can introduce grooves and irregular surface features. In this study, pumice was the least abrasive polishing method for enamel and showed the smallest increase in roughness. Hand instrumentation resulted in a smoother surface than ultrasonic scaling [7].

Morphological analyses revealed microstructural alterations following instrumentation, including superficial scratches, grooves, and localized enamel defects. Manual instrumentation produced an “eroded” surface and partial destruction of enamel prisms. In contrast, ultrasonic scaling caused more significant alterations, such as partial or complete destruction of enamel prism bundles, granular enamel defects, and surface defects. These changes may also relate to vibrational forces during instrumentation, which could disrupt chemical bonds within the enamel. Manual instrumentation was less destructive compared to ultrasonic scaling [20].

### 3.2. Effects on Cementum Surface Roughness

Root surface roughness following periodontal instrumentation was consistently reported, although the degree of surface alterations varied depending on the instrumentation method and study conditions [17,19,21,22,23,24,25,28]. Several studies reported that hand instrumentation was associated with increased surface roughness, grooves, and scratches on the cementum surface [17,21,22,23,25]. SEM analyses showed varying degrees of cementum removal and occasional dentin exposure [17,21,25,26]. In contrast, some researchers found that ultrasonic instrumentation resulted in a smoother, more uniform root surface than manual curettes [21,23,25,26]. Differences were also observed between piezoelectric and magnetostrictive devices, both of which produced smoother surfaces than curettes. Piezoelectric devices created slightly more cracks and substance loss, while magnetostrictive scalers resulted in the least dentin exposure and structural damage [23]. Marda et al. (2012) [26] reported that ultrasonic instrumentation produced the smoothest cementum surfaces, whereas hand instrumentation resulted in scratches and surface irregularities [26]. However, these findings comparing manual with ultrasonic instrumentation were not consistent across all studies [7,25,28].

The power settings of the ultrasonic devices also influenced the outcomes and degree of surface alteration. Medium power was associated with the lowest surface roughness, low power with higher roughness, and high power showed effects comparable to hand instrumentation. Therefore, a positive correlation between ultrasonic power setting and surface alteration was suggested [22].

These findings were also supported by Parashar et al. (2023) [27]. The microscopic examination revealed linear grooves, shallow scratches, and irregularities on the cementum surface following instrumentation. These alterations were attributed to the vibrational motion of the ultrasonic scaler, which damages the root surface. Moreover, the degree of surface alteration was influenced by instrumentation parameters and technique, including applied pressure, tip angulation, and instrumentation duration. Lower pressure and shorter instrumentation time were associated with reduced surface damage, indicating that the effects of periodontal instrumentation are highly operator-dependent. Additionally, worn scaler tips were associated with increased surface roughness compared to new tips [27].

### 3.3. Hard-Tissue Loss and Substance Removal

Cementum loss was consistently reported, but its extent varied [17,19,21,23,26]. Manual curettes were associated with greater removal of root substance during instrumentation [17,21,26]. In the SEM analysis, hand curettes were associated with smoother surfaces but also with the greatest cementum removal, and could lead to complete cementum loss with dentin exposure [3].

In contrast, ultrasonic devices preserved more cementum and caused less tissue loss compared to hand instrumentation [19,21]. However, ultrasonic scaling was not entirely atraumatic, and varying degrees of cementum removal were reported depending on instrumentation parameters [27].

Rotary instruments were associated with greater surface damage than curettes or ultrasonic devices, including increased cementum removal and dentin exposure [26].

Quantitative data from Arefnia et al. [19] measured cementum loss of ≤20 µm after air polishing, ≥20 µm after ultrasonic, and ≥40 µm after hand instrumentation. Air polishing caused no significant loss of substance in enamel, while minimal loss (<3.0 µm) occurred after ultrasonic and hand instrumentation [19].

The extent of root surface alterations and cementum removal was significantly influenced by instrumentation parameters, including increased power settings, longer instrumentation time, and greater lateral force. Worn scaler tips were associated with reduced calculus removal efficiency and increased root surface roughness, potentially leading to greater substance loss due to prolonged instrumentation time and increased mechanical stress on cementum [27].

Bozbay et al. (2018) [3], in an in vivo study with subsequent ex vivo analysis after extraction, reported the highest preservation of cementum with air polishing (94% coronal cementum retained), compared to ultrasonic scaling (84%), ultrasonic scaling followed by air polishing (80%), and hand instrumentation (65%). Similar trends were observed in apical cementum, indicating greater tissue preservation with air-polishing systems [3].

Overall, all investigated instrumentation methods result in some degree of cementum loss, with hand instrumentation generally associated with greater tissue removal, ultrasonic devices showing intermediate effects, and air polishing demonstrating a high level of tissue preservation.

### 3.4. Effects of Air-Polishing Systems

Air polishing was identified as one of the least abrasive techniques for both enamel and cementum [19,24]. Across studies, it was associated with minimal changes in surface roughness and high preservation of hard tissue [3,19,29].

On cementum, air polishing alone produced no significant change in roughness depth compared to the untreated control surfaces, indicating minimal surface alteration [19]. Quantitative data demonstrated that air polishing produced a roughness value (Rz 6.64 µm) closer to that of untreated cementum (Rz 7.48 µm). In contrast, hand instrumentation (3.82 µm) and ultrasonic scaling (2.36 µm) resulted in greater changes in surface roughness [19]. Profilometric analysis confirmed that air polishing resulted in less surface roughness compared to hand instrumentation and can smooth surface irregularities caused by instrumentation [24].

SEM analysis supported these findings, showing general preservation of cementum with only a few grooves and fewer surface defects compared with ultrasonic instrumentation, which produced visible surface irregularities. However, air polishing alone was ineffective at removing calculus. Combining ultrasonic scaling with air polishing appeared effective for preserving cementum and removing calculus [3].

Air-polishing systems had minimal effects on enamel surface roughness and did not result in significant changes compared with untreated enamel surfaces [19,29].

## 4. Discussion

The present narrative review evaluated the effects of various periodontal instrumentation techniques on enamel and cementum surface characteristics, with particular focus on surface roughness and hard-tissue loss. Across the included studies, all examined instrumentation methods showed some degree of surface alteration. However, the outcomes differed in the magnitude of the damage and clinical relevance of these changes.

Surface roughness was consistently reported following periodontal instrumentation. Manual instrumentation was frequently associated with greater surface irregularities and increased cementum removal [20,21,22,23,25]. This may be explained by the direct mechanical action of curettes and the applied lateral pressure used to effectively remove calculus [18]. In contrast, ultrasonic devices generally produced a more uniform root surface [21,23,25,26], and cementum was often mostly intact with minimal surface disruption [26]. This smoother topography is most likely due to high-frequency mechanical vibration of ultrasonic tips rather than the scraping action during manual curettage [31]. Nevertheless, it should be noted that ultrasonic devices were not entirely atraumatic, and surface roughness and microcracks were still observed [22,23].

These findings are supported by Solomon et al. [32], who reported greater hard-tissue loss with hand instrumentation compared to ultrasonic scaling using a weight-based assessment method, which further indicated the more invasive nature of curettes.

Ultrasonic scaling is described as a minimally invasive technique, although it is still associated with surface alterations. The extent of these changes appears to be influenced by operator-dependent variables, such as applied force, angulation, duration of use, or instrument condition. These findings should be interpreted in comparison with other periodontal instrumentation methods, which are also associated with hard-tissue modifications and appear to be operator-dependent [21,27].

Kruse et al. (2024) [29] reported that both curettage and air polishing on enamel produced measurable alterations in surface roughness. Nevertheless, these alterations were described as clinically insignificant, likely due to the high resistance of enamel to periodontal instrumentation, resulting in only minor changes. The researchers also described no measurable enamel loss during instrumentation [29].

Air polishing was described as one of the least-abrasive instrumentation methods for both enamel and cementum [15,21]. It is a conservative approach that effectively removes plaque and stains and offers practical advantages, such as minimizing operator fatigue and accessing anatomically difficult areas that might not be reached during polishing with conventional rubber cups or brushes [24]. However, it is primarily used for supportive periodontal therapy and maintenance, rather than for traditional root planing alone [29,33]. Air polishing after scaling has been shown to be beneficial in in vitro settings due to the “spreading pattern” of air polishing, which may produce a more homogeneous surface topography [19,24]. Nevertheless, the resulting roughness values after air polishing should be interpreted with caution. It was suggested that the higher Rz values observed result from cleaning the depressions occurring in the surface profile, thereby deepening the valleys on the enamel surface. In contrast, the lower values for ultrasonic scaling may indicate a flattening of the naturally occurring peaks [19].

Depending on the treatment objective, different instrumentation techniques resulted in varying in vitro surface characteristics. While effective removal of biofilm and calculus remains essential, preservation of dental hard tissues is emphasized in periodontal therapy [3,33]. Furthermore, roughened enamel and cementum surfaces could enhance plaque retention, microbial colonization, and calculus reformation, potentially compromising long-term periodontal stability and healing [18,26]. However, it should be noted that the observed surface alterations were primarily derived from in vitro studies and may not directly translate into clinically relevant outcomes.

Despite differences in surface alterations, studies have reported comparable calculus removal effectiveness with hand and ultrasonic instruments [23,25,26]. Both techniques significantly reduced subgingival deposits, with no statistically significant differences in debridement efficacy [23].

When choosing a debridement technique, treatment time and operator-related factors should also be considered. Ultrasonic instrumentation required significantly less working time than manual scaling. Reduced treatment duration is most likely associated with lower operator fatigue [26]. Manual curettage offers a better tactile sense but has been described as technique-sensitive, time-consuming, and physically demanding for the operator [18].

The ideal instrumentation approach was defined as one that effectively removes plaque, calculus, and endotoxins while preserving cementum and minimizing iatrogenic damage [26].

The majority of included studies were conducted in vitro on extracted teeth and were free of caries or structural defects [18,22,26]. Moreover, in vitro settings do not fully replicate the biological environment of the oral cavity, which may affect the long-term prognosis of periodontal treatment [28].

Heterogeneity was observed across studies regarding instrumentation parameters, operator techniques, measurement methods, and outcome reporting. This variability limits direct comparability and complicates interpretation of overall trends [18,19,22,26].

Comparing surface roughness outcomes across studies was complicated by the different measurement techniques. Scanning electron microscopy (SEM), atomic force microscopy (AFM), and profilometry, which were primarily used, assess surface characteristics at different spatial scales and at various levels of resolution. Furthermore, studies report different roughness parameters, such as average roughness (Ra) or roughness depth (Rz), which are not directly comparable. While Ra reflects the mean deviation, Rz describes peak-to-valley differences and may therefore show higher roughness values for the same surface [7,19,24,25].

Another difficulty in comparison was that the concept of a “smooth surface” was inconsistently defined across studies, since lower roughness values do not necessarily indicate an improved outcome. Surface smoothing may also result from the removal of cementum rather than from the preservation of tissue integrity [7,19].

Additionally, this review was conducted as a narrative synthesis, and no formal risk-of-bias assessment was performed. The methodological quality and internal validity of the included studies vary due to differences in experimental design and a lack of standardization. Furthermore, the external validity of in vitro study designs is limited because laboratory conditions may not fully reflect clinical practice. The study selection process may also be subject to selection bias. These factors should be considered when interpreting the strength and applicability of the presented evidence.

Overall, periodontal instrumentation is used to remove plaque, calculus, and endotoxins while preserving the dental hard tissues [7], since extensive removal of cementum could compromise periodontal healing. Preservation of cementum was emphasized to achieve a positive outcome in periodontal therapy. From a clinical perspective, periodontal instrumentation is highly important because maintaining a clean tooth surface and reducing bacterial load may help limit the progression of periodontitis [7].

The findings of this review suggest that current instrumentation techniques are effective in biofilm and calculus removal, but they differ in their impact on enamel and cementum. These differences may be biologically relevant because surface roughness or excessive removal of cementum could potentially influence plaque accumulation and periodontal healing [34]. However, a direct clinical translation of these results remains uncertain. At the same time, an overly conservative approach may compromise effective debridement, suggesting that an appropriate balance between effective biofilm removal and preservation of hard tissues is essential.

An ideal instrumentation approach has been described as one that effectively removes plaque, calculus, and endotoxins while preserving cementum and minimizing iatrogenic damage [26]. No single instrumentation system currently fulfils all of these requirements [19].

Future research should prioritize standardized methodologies and clinically oriented study designs to better evaluate the long-term outcomes associated with different periodontal instrumentation techniques.

## 5. Conclusions

Within the given limitations of the evaluated preclinical evidence, periodontal instrumentation was associated with alterations to enamel and cementum surfaces. Manual instrumentation was associated with greater surface irregularities and increased cementum removal, whereas ultrasonic instrumentation was associated with more uniform surface characteristics and comparatively lower substance loss. Air-polishing systems were described as relatively less invasive when used for biofilm removal and polishing in laboratory settings.

The findings of this review require a cautious interpretation. Most of the included studies were conducted in vitro and showed considerable heterogeneity in study design, instrumentation parameters, and outcome assessment. Consequently, the clinical relevance of the observed surface alterations remains uncertain.

## Figures and Tables

**Table 1:** Summary of the included studies on the effects of periodontal instrumentation on enamel and cementum.

Study	Study Design	Sample Size	Substrate	Instrumentation	Comparator	Measurement Method	Outcome	Key Finding
Marda et al., 2012 [26]	In vitro	*n* = 36	Cementum	Manual Ultrasonic Rotary	Comparison between groups	SEM	No significant difference in calculus removal or tooth substance loss between groups; Significant difference in surface roughness; Ultrasonic showed the lowest roughness.	Ultrasonic instrumentation produced smoother root surfaces and less hard-tissue loss compared to hand and rotary instruments.
Parashar and Bhavsar, 2023 [27]	In vitro	*n* = 160	Cementum	Piezoelectric ultrasonic scaling	New and worn scaler tips under varying parameters (Power setting, lateral force, and time)	Contact surface profilometer, AFM	Surface roughness increased after instrumentation in all groups; Worn tips produced significantly higher roughness than new tips; Increased lateral force, power, and time increased cementum roughness.	Scaler tip wear significantly increases root surface roughness, with lateral force and instrumentation time being the most influential factors. Worn scaler tips consistently produced rougher surfaces than new tips.
Dahiya et al., 2011 [21]	In vitro	*n* = 20	Cementum	Manual Ultrasonic Rotary	Comparison between groups 2 controls (no instrumentation)	SEM	Mean Roughness and Loss of Tooth Substance Index scores were the highest for hand instruments, and lowest for rotary instruments. Significant differences between rotary and the other two groups; No significant difference between curettes and ultrasonic instrumentation.	Rotary instruments produced the smoothest root surfaces with the least apparent roughness, while hand curettes caused the greatest surface roughness and hard-tissue loss; ultrasonic instruments showed intermediate effects.
Zafar, 2016 [28]	In vitro	*n* = 40	Cementum	Manual Ultrasonic	Comparison between groups, Control (before treatment)	Non-contact profilometry, nanoindenter	Significant reduction in surface roughness after both instrumentation techniques, with no significant increase in elastic modulus.	Both methods reduced roughness. Manual instrumentation produced smoother surfaces than ultrasonic, but the difference was not statistically significant.
Kumar et al., 2015 [22]	In vitro	*n* = 35	Cementum	Manual Ultrasonic	Comparison between control (no instrumentation), manual, and ultrasonic (three power settings) groups	3D optical profilometer, Field Emission Scanning Electron Microscopy (FESEM)	Highest roughness: ultrasonic low power setting; Intermediate roughness: manual curettage and ultrasonic high-power setting; Lowest roughness: ultrasonic medium power setting.	Instrumentation increased surface roughness compared to the control group; ultrasonic at medium power setting produced the smoothest surfaces; hand instruments showed comparable roughness to high-power ultrasonic setting.
Al Ankily et al., 2020 [18]	In vitro	*n* = 40	Enamel	Ultrasonic—stainless steel Ultrasonic—titanium nitride Manual—stainless steel Manual—titanium	Comparison between hand and ultrasonic Comparison between titanium and stainless steel, as well as between groups	AMF, SEM	Surface roughness increased in all groups; highest with hand titanium, lowest with ultrasonic stainless steel.	Ultrasonic stainless-steel tips caused the least surface roughness and damage. Hand instruments and titanium tips caused significantly greater roughness and more destructive surface changes.
Arefnia et al., 2021 [19]	In vitro	*n* = 5 (five untreated reference surfaces and 45 treatment surfaces)	Enamel and Cementum	Air polishing Ultrasonic Manual Rubber-cup polishing	Comparison between untreated control and multiple intergroup comparisons (different techniques and combinations)	Optical microcoordination measuring	Surface roughness and substance loss varied by method; Air polishing caused minimal or no enamel loss and the least cementum loss; Hand and ultrasonic caused greater substance loss.	Air polishing (nonabrasive powder) showed the best hard-tissue preservation. Combining techniques increased abrasion and offered no benefit.
Sultan et al., 2022 [24]	In vitro	*n* = 45	Cementum	Manual Manual in combination with air polishing Air polishing alone	Comparison between groups	Profilometry	Surface roughness was the highest with hand instrumentation and the lowest with air polishing alone.	Air polishing can even out surface irregularities caused by and or ultrasonic instrumentation.
Bozbay et al., 2018 [3]	In vivo (with ex vivo analysis)	*n* = 48	Cementum	Manual Ultrasonic Ultrasonic in combination with air polishing Air polishing alone	Instrumented surface compared to non-instrumented surface; Comparison between groups	Histological analysis, SEM	Cementum loss in all groups. Hand instrumentation: highest substance loss Air polishing: lowest Ultrasonic: intermediate.	Air polishing preserved the most cementum; hand instruments caused the greatest hard-tissue loss while producing the smoothest surface; ultrasonic instruments caused grooves and scratches, which were also observed after performing air polishing.
Kruse et al., 2024 [29]	Ex vivo	*n* = 640	Enamel and Dentin	Manual Air polishing Rubber cup polishing Combinations	Negative control (pellicle only) and baseline (pretreatment), comparison between groups	Profilometry	No measurable enamel loss was observed.	All methods caused only minor changes in enamel roughness of limited clinical relevance; none caused enamel loss; polishing offers no benefit.
Kulygina et al., 2025 [20]	In vivo (with ex vivo analysis)	*n* = 8	Enamel, Cementum, and Dentin	Manual Ultrasonic Sonic scaling	Comparison between groups	Morphological analysis	All methods caused structural surface alterations. Manual scaling caused surface irregularities and left residual deposits. Ultrasonic and sonic caused destructive changes in enamel prisms; sonic appeared most aggressive; cementum partially destroyed (especially with sonic).	Sonic and ultrasonic scaling caused structural damage (enamel prism destruction, “eroded” surfaces); Hand scaling was less destructive but incomplete deposit removal; All methods resulted in uneven surfaces.
Wasti et al., 2023 [25]	In vivo (with ex vivo analysis)	*n* = 90	Cementum	Manual Ultrasonic	Control (no instrumentation), comparison between groups	SEM	Surface roughness was the highest in the untreated control group, lower with hand, and lowest with ultrasonic. Both methods caused surface alterations.	Ultrasonic scaling produced smoother root surfaces than hand instrumentation or the control group. Both methods were effective in calculus removal.
Yaghini et al., 2015 [30]	In vitro	*n* = 56	Cementum	Er: YAG laser Manual Ultrasonic	Control group (no instrumentation), comparison between groups	Profilometry	Surface roughness is highest with the laser, intermediate with the hand, and lowest with the ultrasonic. The difference between laser and ultrasonic was significant. No significant difference between ultrasonic and the control group. Comparison among other groups showed no significant difference.	Ultrasonic instrumentation produced the smoothest root surfaces; Er: YAG laser resulted in higher roughness and offered no advantage over hand instrumentation.
Yildirim et al., 2021 [7]	In vitro	*n* = 200	Enamel and Cementum	Manual Ultrasonic Polishing (paste, pumice, air powder)	Baseline (pretreatment), comparison between groups	Profilometry	Enamel: Surface roughness increased in all groups; the highest increase was with ultrasonic, the lowest with polishing (especially pumice); hand instruments produced less roughness than ultrasonic. Cementum: Surface roughness increased in all groups. The highest increase was with ultrasonic.	Ultrasonic scaling caused the greatest increase in roughness on both enamel and root surfaces; polishing did not restore baseline smoothness in enamel or cementum.
Mittal et al., 2014 [23]	In vivo (with ex vivo analysis)	*n* = 40	Cementum	Ultrasonic Manual	Control (no instrumentation), comparison between groups	Profilometry, SEM, Planimetric analysing tool	Hand instrumentation: deep scratches and gouges; ultrasonic: smoother surfaces but still irregular. Deposit removal was similar in all groups.	Hand instrumentation produced the roughest surfaces and greatest structural damage. Ultrasonic devices produced smoother surfaces but still caused substance loss.
Dahiya & Kamal, 2012 [17]	In vitro	*n* = 20	Cementum	Manual Ultrasonic Rotary bur	Control (no instrumentation), comparison between groups	SEM	Hand instrumentation showed the highest roughness; less roughness was seen with ultrasonic and lowest with rotary; hand instrumentation caused deep scratches and significant tissue loss; all techniques effectively removed deposits.	Rotary instruments produced the smoothest surfaces with the least damage; hand instruments caused the greatest roughness and substance loss.

## Data Availability

No new data were created or analysed in this study. Data sharing is not applicable to this article.

## Abbreviations

The following abbreviations are used in this manuscript:

AFM	Atomic Force Microscopy
SEM	Scanning Electron Microscopy
SPT	Supportive Periodontal Therapy

## References

[1] Manresa C., Sanz-Miralles E.C., Twigg J., Bravo M. (2018). Supportive Periodontal Therapy (SPT) for Maintaining the Dentition in Adults Treated for Periodontitis. Cochrane Database Syst. Rev..

[2] Van Dyke T.E., Sheilesh D. (2005). Risk Factors for Periodontitis. J. Int. Acad. Periodontol..

[3] Bozbay E., Dominici F., Gokbuget A., Cintan S., Guida L., Aydin M., Mariotti A., Pilloni A. (2018). Preservation of Root Cementum: A Comparative Evaluation of Power-driven versus Hand Instruments. Int. J. Dent. Hyg..

[4] Nyman S., Westfelt E., Sarhed G., Karring T. (1988). Role of “Diseased” Root Cementum in Healing Following Treatment of Periodontal Disease. A Clinical Study. J. Clin. Periodontol..

[5] Paramashivaiah R., Prabhuji M.L.V. (2013). Mechanized Scaling with Ultrasonics: Perils and Proactive Measures. J. Indian Soc. Periodontol..

[6] Kumari N., Johnson L., Yadav H., Das A., Umrao B., Gera R. (2023). Effect of Hand and Ultrasonic Scaling-Root Planing Methods on Tooth Surface Topography: An In-Vitro Atomic Force Microscopy Study. Cureus.

[7] Yildirim T.T., Oztekin F., Keklik E., Tozum M.D. (2021). Surface Roughness of Enamel and Root Surface after Scaling, Root Planning and Polishing Procedures: An in-Vitro Study. J. Oral. Biol. Craniofacial Res..

[8] Kwon T., Lamster I.B., Levin L. (2021). Current Concepts in the Management of Periodontitis. Int. Dent. J..

[9] Beniash E., Stifler C.A., Sun C.-Y., Jung G.S., Qin Z., Buehler M.J., Gilbert P.U.P.A. (2019). The Hidden Structure of Human Enamel. Nat. Commun..

[10] Lampe Bless K., Sener B., Dual J., Attin T., Schmidlin P.R. (2011). Cleaning Ability and Induced Dentin Loss of a Magnetostrictive Ultrasonic Instrument at Different Power Settings. Clin. Oral. Investig..

[11] Abdul Hayei N.A., Yahya N.A., Safii S.H., Saub R., Vaithilingam R.D., Baharuddin N.A. (2021). Influence of Scaler Tip Design on Root Surface Roughness, Tooth Substance Loss and Patients’ Pain Perception: An in Vitro and a Randomised Clinical Trial. BMC Oral. Health.

[12] Babina K., Polyakova M., Sokhova I., Doroshina V., Arakelyan M., Zaytsev A., Novozhilova N. (2021). The Effect of Ultrasonic Scaling and Air-Powder Polishing on the Roughness of the Enamel, Three Different Nanocomposites, and Composite/Enamel and Composite/Cementum Interfaces. Nanomaterials.

[13] Janakiram C., Dye B.A. (2000). A Public Health Approach for Prevention of Periodontal Disease. Periodontology.

[14] Tan A. (2009). Periodontal Maintenance. Aust. Dent. J..

[15] Ossom Williamson P., Minter C.I.J. (2019). Exploring PubMed as a Reliable Resource for Scholarly Communications Services. J. Med. Libr. Assoc..

[16] Sabatini S., Maiorani C., Bassignani J., Cotellessa S., Di Trani G., Fulgenzi E., Iacono R., Mercogliano I., Butera A. (2024). Effectiveness of Ultrasonic and Manual Instrumentation in Nonsurgical Periodontal Therapy: Are Additional Therapies More Effective? A Systematic Review. Appl. Sci..

[17] Dahiya P., Kamal R. (2012). Ultra-Morphology of Root Surface Subsequent to Periodontal Instrumentation: A Scanning Electron Microscope Study. J. Indian Soc. Periodontol..

[18] Al Ankily M., Makkeyah F., Bakr M., Shamel M. (2020). Effect of Different Scaling Methods and Materials on the Enamel Surface Topography: An in Vitro SEM Study. J. Int. Oral. Health.

[19] Arefnia B., Koller M., Wimmer G., Lussi A., Haas M. (2021). In Vitro Study of Surface Changes Induced on Enamel and Cementum by Different Scaling and Polishing Techniques. Oral. Health Prev. Dent..

[20] Kulygina V.M., Arshynnikov R.S., Drohomyretska M.S., Pylypiuk O.Y., Gadzhula N.G., Poberezhna H.M., Polyanyk N. (2025). Morphological Studies of Changes in Dental Hard Tissues Following Different Methods of Dental Deposit Removal. Wiad. Lek..

[21] Dahiya P., Kamal R., Gupta R., Pandit N. (2011). Comparative Evaluation of Hand and Power-Driven Instruments on Root Surface Characteristics: A Scanning Electron Microscopy Study. Contemp. Clin. Dent..

[22] Kumar P., Das S.J., Sonowal S.T., Chawla J. (2015). Comparison of Root Surface Roughness Produced By Hand Instruments and Ultrasonic Scalers: An Invitro Study. J. Clin. Diagn. Res..

[23] Mittal A., Nichani A., Venugopal R., Rajani V. (2014). The Effect of Various Ultrasonic and Hand Instruments on the Root Surfaces of Human Single Rooted Teeth: A Planimetric and Profilometric Study. J. Indian Soc. Periodontol..

[24] Sultan F., Joshi N., Rathod V. (2022). In Vitro Analysis of Surface Roughness Produced by an Air Polishing Device and Conventional Root Planing on Cementum: A Profilometric Study. J. Indian Soc. Periodontol..

[25] Wasti J., Ravishankar P.L., Wasti A., Rajula M.P.B., Sunanda K., Alzahrani K.J., Alharif K.F., Halawani I.F., Alzahrani F.M., Baeshen H.A. (2023). Root Surface Changes Following Manual and Ultrasonic Instrumentation—A Scanning Electron Microscopic Study. Eur. Rev. Med. Pharmacol. Sci..

[26] Marda P., Prakash S., Devaraj C., Vastardis S. (2012). A Comparison of Root Surface Instrumentation Using Manual, Ultrasonic and Rotary Instruments: An in Vitro Study Using Scanning Electron Microscopy. Indian J. Dent. Res..

[27] Parashar A., Bhavsar N. (2023). Assessing the Effect of Piezoelectric Ultrasonic Scaler Tip Wear on Root Surface Roughness under Influence of Various Working Parameters: A Profilometric and Atomic Force Microscopic Study. J. Indian Soc. Periodontol..

[28] Zafar M.S. (2016). Comparing the Effects of Manual and Ultrasonic Instrumentation on Root Surface Mechanical Properties. Eur. J. Dent..

[29] Kruse A.B., Fortmeier S., Vach K., Hellwig E., Ratka-Krüger P., Schlueter N. (2024). Impact of Air-Polishing Using Erythritol on Surface Roughness and Substance Loss in Dental Hard Tissue: An Ex Vivo Study. PLoS ONE.

[30] Yaghini J., Naghsh N., Attaei E., Birang R., Birang E. (2015). Root Surface Roughness After Scaling and Root Planing with Er:YAG Laser Compared to Hand and Ultrasonic Instruments by Profilometry. J. Dent..

[31] You X., Wu X., Chen S. (2024). Effects of a New Magnetostrictive Ultrasonic Scaler and a Traditional Piezoelectric Ultrasonic Scaler on Root Surfaces and Patient Complaints. Sci. Rep..

[32] Solomon S.M., Stoleriu S., Forna D.A., Tampu D., Stefanache M.A.M., Ursarescu I.G., Martu S. (2016). The Quantitative and Qualitative Assessment of Dental Substance Loss as Consequence of Root Planing by Three Different Techniques. Mater. Plast..

[33] Caygur A., Albaba M.R., Berberoglu A., Yilmaz H.G. (2017). Efficacy of Glycine Powder Air-Polishing Combined with Scaling and Root Planing in the Treatment of Periodontitis and Halitosis: A Randomised Clinical Study. J. Int. Med. Res..

[34] Janik K., Skucha-Nowak M. (2025). Root Cementum Molecular Structure and Its Role in Maintaining Oral Health-Systematic Review. Int. J. Mol. Sci..

